# A comparative study on the antifungal effect of potassium sorbate, chitosan, and nano-chitosan against *Rhodotorula mucilaginosa* and *Candida albicans* in skim milk acid-coagulated (Karish) cheese

**DOI:** 10.14202/vetworld.2023.1991-2001

**Published:** 2023-09-23

**Authors:** Shimaa S. Awaad, Marwa A. Sherief, Sahar M. Mousa, A. Orabi, Ayah B. Abdel-Salam

**Affiliations:** 1 Department of Food Hygiene and Control, Faculty of Veterinary Medicine, Cairo University, Giza, Egypt; 2 Inorganic Chemistry Department, Advanced Materials Technology and Mineral Resources Research Institute, National Research Centre, Giza, Egypt; 3 Department of Microbiology, Faculty of Veterinary Medicine, Cairo University, Giza, Egypt

**Keywords:** *Candida*, chitosan, nano-chitosan, Karish cheese, potassium sorbate, *Rhodotorula*

## Abstract

**Background and Aim:**

Yeasts are common contaminants in the cheese industry, which frequently arise from raw milk, the surrounding environment, and equipment, resulting in economic losses in addition to health hazards. This study aimed to compare the antifungal effect of chitosan and nano-chitosan as natural preservatives with a commonly used chemical preservative (potassium sorbate) against *Rhodotorula mucilaginosa* and *Candida albicans*.

**Materials and Methods:**

Laboratory Karish cheese was manufactured with the addition of potassium sorbate, chitosan, nano-chitosan, and their combinations at different concentrations. The survival of *R. mucilaginosa* and *C. albicans* was monitored in different treatments (CR, PR1, PR2, CR1, CR2, NR1, NR2, MR, CC, PC1, PC2, CC1, CC2, NC1, NC2, MC) during storage in a refrigerator with continuous measurement of pH. The impact of using these antifungal agents on the organoleptic parameters of Karish cheese during storage was also evaluated.

**Results:**

There was a significant decrease in the count of yeasts in all treatments from the 3^rd^ day of storage, while the mixture of 0.1% potassium sorbate (MR) and 2% chitosan (MC) improved the antifungal effect of chitosan with a lower potassium sorbate concentration and showed the best antifungal effects against both *R. mucilaginosa* and *C. albicans*. This combination reduced the yeast count from 8.92 and 9.57 log_10_ colony-forming unit (CFU)/g in MR and MC treatments, respectively, until it became undetectable on the 9^th^ day of storage, which was earlier than for all other treatments. It was noted that the addition of chitosan nanoparticles (ChNPs) at either 0.25% (NR1 and NC1) or 0.5% (NR2 and NC2) during the manufacturing of Karish cheese significantly lowered the counts of *R. mucilaginosa* and *C. albicans* compared with chitosan with a higher molecular weight, but significantly lower than potassium sorbate until 6^th^ day of storage as all treatments of chitosan nanoparticles became significantly higher than potassium sorbate treatments. After 9 days of storage, NR2 and NC2 treatments showed the most significant decreases in count (3.78 and 4.93 log_10_ CFU/g, respectively), indicating better stability of ChNPs. At the end of the storage period, PR2, PC2, CR2, and CC2 showed significantly high pH values among the groups of 4.8, 5.0, 4.8, and 5.1, respectively. The overall acceptability was significantly higher in treated Karish cheese samples than in the control group, especially at the end of the storage period.

**Conclusion:**

Potassium sorbate, chitosan, and ChNPs are effective antifungal preservatives against *R. mucilaginosa* and *C. albicans*. In addition, the combination of chitosan with potassium sorbate showed synergistic antifungal activity. These additives also preserve the sensorial criteria longer than for cheese without preservatives.

## Introduction

Cheese is a fundamental part of the daily diet in Egypt, which is sometimes consumed as often as 3 times a day. Several types of white soft cheese are produced locally in Egypt in different regions using traditional recipes. Karish cheese can be considered one of the most popular local types of fresh soft cheese among Egyptian consumers [1]. Egypt has the highest cheese consumption rate in North Africa, with a wide variety of types, including soft, semi-soft, and hard cheeses with different salt contents. The most popular types of Egyptian cheeses are Domiati, Karish, and Ras [2].

Karish cheese is a soft, white curd, acid-coagulated fresh cheese with a somewhat salty flavor that is manufactured from skim milk cow or buffalo milk. It is one of the most common food products high in protein, calcium, and phosphorus with low-fat content, which is affordable, making it the most popular type among the traditional cheeses. It also has an abundance of potassium salts, which are crucial for developing body fluids and muscles [3].

The traditional method of producing Karish cheese is associated with a high risk of microbial contamination through several routes, including dependance on raw milk with poor bacteriological quality, unsterilized production conditions, and not being covered when on sale. Furthermore, it can be considered as a suitable medium for microbial growth [4].

Yeast is generally recognized as a contaminant in the cheese industry. The most common yeast species are *Debaryomyces hansenii*, *Geotrichum candidum*, *Kluyveromyces marxianus*, *Kluyveromyces lactis*, *Rhodotorula mucilaginosa*, *Candida* spp., and *Trichosporon* spp. The degree of yeast spoilage depends on the species and strain levels. The excessive growth of yeast in cheese is considered a microbiological hazard and affects cheese quality [5].

The growth of yeast on cheese surfaces at a rate exceeding 10^6^ colony-forming units (CFU)/g leads to the appearance of red or yellow spots, wrinkled peel, darkened appearance, slimy texture, and various other defects. The production of various yeast metabolites results in abnormalities in cheese flavor, such as yeasty, alcoholic, fruity, rancid, lard-like, and other foreign flavors, thereby reducing the cheese quality. In addition, some yeast strains can generate carbon dioxide (CO_2_), which may result in the formation of holes within cheese blocks “early blowing faults”, self-splitting, and a spongy texture [6]. Some species of the genus *Rhodotorula* cause staining and confer a bitter taste on cheese during storage [7].

However, a number of yeasts metabolize the organic acids in fermented foods, raising the pH and promoting the growth of pathogenic and spoiling bacteria. Some yeasts may further threaten food safety, given their link with opportunistic infections and other adverse conditions in humans [3].

Due to the shelf-life of fresh cheese is very short, ranging between 1 and 2 weeks [8] with a maximum expiration period of 1 month, according to the Egyptian Standards for white soft cheese [9], there are numerous opportunities to prevent microbial contamination during the conventional production process.

These opportunities include the observation of sanitary and hygienic guidelines, efficient equipment cleaning and disinfection, air filtration, ozonization of premises, and improving awareness about food safety concepts, including good manufacturing practices and hazard/critical control points. Physical measures for yeast control are another option, such as the heat treatment of milk and brine, storage in a refrigerator (about 4°C), and the microfiltration process for milk and cheese whey. Chemical control measures are also available, including the use of preservatives (natamycin and potassium sorbate) and the packaging of cheese in aseptic conditions using modified gases (50%–90% CO_2_) or vacuum packaging [6]. The use of selected starter cultures with fungicide properties as a biological control measure is also possible [3].

The European Community Regulations [10] define food additives and preservatives as “substances which prolong the shelf-life of foods by preserving them against spoilage caused by microorganisms and/or which guard against growth of pathogens.” The addition of preservatives during cheese processing, such as sorbic acid, sodium benzoate, hydrogen peroxide, nisin, natamycin, and chitosan, is one of the simplest ways to extend the shelf-life of final cheese products and also to retard the physical alterations caused by spoilage microorganisms [11].

Potassium sorbate (E202) is considered the most widely used food preservative around the world because it is regarded as a “Generally Recognized as Safe” (GRAS) food additive and is much more soluble in water than sorbic acid. It is effective up to pH 6.5, but its effectiveness increases with decreasing pH. Potassium sorbate is effective against yeast, mold, and some bacteria, and should be added to cheese at a rate of 0.025%–0.1% [12]. It was listed in the Union list of food additives established by the Commission Regulation [13] as an antifungal preservative for fresh and ripened cheese.

Chitosan is a natural biopolymer consisting of glucosamine and N-acetylglucosamine residues with a 1, 4-β-linkage. It is the world’s second most prevalent natural polymer after cellulose due to its outstanding biodegradability, biocompatibility, antimicrobial activity, nontoxicity, and economic benefits. Therefore, it was added to the GRAS list by the US Food and Drug Administration in 2011 and regarded as one of the most promising materials for future uses as a food preservative [14, 15]. The previous study by Radhakrishnan *et al*. [16] suggested that the antimicrobial activity of chitosan is modified by acidic conditions and reduced at neutral pH. A wide range of applications of chitosan as an antimicrobial agent in dairy products have been reported [17–21].

The antimicrobial activity of chitosan depends on its molecular weight (MW); therefore, the size of chitosan is the main factor affecting its activity. In this context, nano-chitosan was prepared and used in this study. Several methods have been developed to prepare chitosan nanoparticles (ChNPs), namely, an emulsion method, ionic gelation method [22–24], reverse micellar method, and self-assembling method [25, 26]. This research considers the ionic gelation method due to its simplicity and low cost. This study aimed to compare the antifungal effects of chitosan with a regular MW and nano-chitosan as natural, safe substitute for chemical preservative (potassium sorbate) against certain spoilage yeasts (*Rhodotorula* spp. and *Candida* spp.) in white soft Karish cheese during cold storage.

## Materials and Methods

### Ethical approval

Ethical approval was not required for this study. All the experiments were performed *in vitro*.

### Study period and location

This study was conducted from January to May 2023 at the Laboratory of Food Hygiene and Control, Faculty of Veterinary Medicine, Cairo University, Egypt.

### Materials

Fresh buffalo’s skim milk (0.5% fat and 8.5% solid not fat) was purchased from the Faculty of Agriculture farm, Cairo University, Egypt. Freeze dried-direct vat set with *Lactobacillus delbrueckii* spp. *Bulgaricus*, *Lactococcus lactis* spp. *Lactis*, *L. lactis* spp. *Cremoris*, and *Streptococcus thermophilus* was purchased as commercial starter culture from Chr. Hansen (White Daily 41). Two reference strains of *R. mucilaginosa* (ATCC 14579) and *Candida albicans* (ATCC 5679) previously isolated and identified from dairy products were used; the stocks were stored frozen at −80°C ± 2°C. These strains were obtained from Cairo-MIRCEN, Faculty of Agriculture, Ain-Shams University. Food-grade fine salt was obtained from El-Nasr Salines Company, Egypt. Calcium chloride was obtained from Sigma Chemical Company, Str. Louis, USA. Microbial rennet powder (Reniplus 2000 IMCU from Caglio Star, Proquiga, Spain). Potassium sorbate, produced by Z.K.W. China, was obtained from Gersy Commercial Co. “Alex.”, Egypt. Chitosan was purchased from Alfa Aesar Company (China), degree of deacetylation (DD) was 85%, MW (1526.464 g/moL).

### Preparation of ChNPs

**Figure-1:**
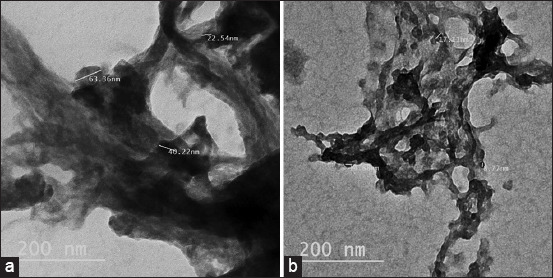
Transmission electron microscopy images of (a) commercial chitosan and (b) prepared chitosan nanoparticles.

Nano-chitosan was prepared using the ionotropic gelation method [27, 28], where 0.5 g of chitosan was dissolved in 1% v/v acetic acid solution and the mixture was stirred at 30°C until it became clear. A total of 100 mL of sodium tripolyphosphate aqueous solution with a concentration of 0.25% w/v was added dropwise under a vigorous magnetic stirrer (69´ *g*). The pH of the mixture was adjusted to 8.5 by adding sodium hydroxide solution (1.0 M). The solution containing ChNPs was decanted several times using distilled water until its pH became neutral, to eliminate any excess sodium hydroxide. The nano-chitosan was filtered by centrifugation and then freeze-dried for analysis and use (Figure-1).

### Strain preparation

*Rhodotorula mucilaginosa* and *C. albicans* were activated in Sabouraud broth (HiMedia, LQ120D, India) supplemented with 100 mg/L chloramphenicol and incubated at 25°C for 48 h to reach a final concentration of approximately 8 log_10_ CFU/mL, which was counted by a plating method on Sabouraud dextrose agar (SDA) (HiMedia) plates supplemented with 100 mg/L chloramphenicol. One milliliter of the culture was serially diluted in 1% peptone water to attain the desired inoculum levels.

### Karish cheese preparation

Thirteen liters of fresh milk was pasteurized in the laboratory at 74°C for 15 s, after which it was quickly cooled to 37°C to add starter culture, rennet, and calcium chloride, as per the manufacturer’s instructions. The milk was allowed to coagulate for 40–45 min, after which the curd was scooped into mats similar to those used conventionally to resemble the traditional appearance of Karish cheese. The surface of the curd was then sprinkled with dry salt (2.5 g/100 g cheese) and left to drain. The completed cheese was cut into pieces of a suitable size for each examination interval and stored in sealed plastic containers immersed completely in its salted whey at 4°C [29].

### Effects of potassium sorbate, chitosan, nano-chitosan, and their combinations on the growth of *R. mucilaginosa*

Nine samples of Karish cheese were prepared as in the previously mentioned Karish cheese preparation step with the inoculation of *R. mucilaginosa* at approximately 11 log_10_ CFU/mL milk. The first one was the control sample, which was inoculated only with *R. mucilaginosa* without any antifungal agents (CR). The other samples were inoculated with *R. mucilaginosa* in addition to several used antifungal agents at different concentration levels, as indicated in Table-1. All samples were periodically examined at 0, 3, 6, 9, 12, 15, 18, and 21 days, or until the appearance of signs of spoilage (s) to determine the survival rate of *R. mucilaginosa* as well as the pH. The concentrations used were those recommended in the previous studies by Awaad *et al*. [12], El-Diasty *et al*. [14], Radhakrishnan *et al*. [16], Shawkat *et al*. [28], Stopforth *et al*. [30] Yavuz and Korukluoglu [31] as the best concentrations for achieving antifungal effects.

**Table-1:** Karish cheese-prepared samples inoculated with *R. mucilaginosa* and supplemented with different antifungal agents and their concentrations.

Symbol	Explanation
CR	Control sample inoculated only with *R. mucilaginosa.*
PR1	Test sample inoculated with *R. mucilaginosa* and supplemented with 0.1% potassium sorbate.
PR2	Test sample inoculated with *R. mucilaginosa* and supplemented with 0.2% potassium sorbate.
CR1	Test sample inoculated with *R. mucilaginosa* and supplemented with 1% chitosan.
CR2	Test sample inoculated with *R. mucilaginosa* and supplemented with 2% chitosan.
NR1	Test sample inoculated with *R. mucilaginosa* and supplemented with 0.25% nano-chitosan.
NR2	Test sample inoculated with *R. mucilaginosa* and supplemented with 0.5% nano-chitosan.
MR	Test sample inoculated with *R. mucilaginosa* and supplemented with a mixture of 0.1% potassium sorbate and 2% of chitosan.

*R. mucilaginosa*=*Rhodotorula mucilaginosa.*

### Effect of potassium sorbate, chitosan, nano-chitosan, and their combinations on the growth of *C. albicans*

Another nine samples of Karish cheese were prepared and examined as in part 1a, but with the inoculation of *C. albicans* at approximately 10 log_10_ CFU/mL milk. The first one was the control sample, which was only inoculated with *C. albicans* without any antifungal agents (CC). The other samples were inoculated with *C. albicans* in addition to several used antifungal agents at different concentration levels, as indicated in Table-2.

**Table-2:** Karish cheese-prepared samples inoculated with *C. albicans* and supplemented with different antifungal agents and their concentrations.

Symbol	Explanation
CC	Control sample inoculated only with *C. albicans.*
PC1	Test sample inoculated with *C. albicans* and supplemented with 0.1% potassium sorbate.
PC2	Test sample inoculated with *C. albicans* and supplemented with 0.2% potassium sorbate.
CC1	Test sample inoculated with *C. albicans* and supplemented with 1% chitosan.
CC2	Test sample inoculated with *C. albicans* and supplemented with 2% chitosan.
NC1	Test sample inoculated with *C. albicans* and supplemented with 0.25% nano-chitosan.
NC2	Test sample inoculated with *C. albicans* and supplemented with 0.5% nano-chitosan.
MC	Test sample inoculated with *C. albicans* and supplemented with a mixture of 0.1% potassium sorbate and 2% of chitosan.

*C. albicans=Candida albicans.*

### Impact of using potassium sorbate, chitosan, and nano-chitosan on the organoleptic parameters of Karish cheese

Four separate Karish cheese control samples were prepared as in the previously mentioned steps for cheese preparation and supplemented with 0.1% potassium sorbate (P1), 2% chitosan (C2), 0.5% nano-chitosan (N2), or the combination of 0.1% potassium sorbate and 2% chitosan (M) to determine the sensorial acceptability of the different treatments, compared with another control Karish cheese sample prepared in the traditional way without any supplement (C).

### Examination of prepared Karish cheese samples

#### Microbiological examination

The survival rates of *R. mucilaginosa* and *C. albicans* were determined at the previously mentioned intervals using a spreading technique on SDA plates, as recommended previously [32].

#### Sensory examination

In accordance with the American Dairy Science Association scorecard scheme [33], a sensory examination was conducted in which panelists were asked to give an evaluation score as follows: Flavor (10 points), body and texture (5 points), and color and appearance (5 points). The overall score (out of 20) was calculated as a percentage. Seven experienced sensory panelists (from both sexes in the age range of 30–50 years) were selected from among staff members of the Food Hygiene and Control Department at the Faculty of Veterinary Medicine, Cairo University, Egypt. The prepared Karish cheese samples were cut, placed on plates, and presented to the panelists at random.

#### Chemical examination

pH values were determined for all samples at the described intervals using a waterproof pH instrument (AD11; Adwa^®^, Szeged, Hungary), in accordance previous study by AOAC [34].

### Morphological properties of chitosan

High-resolution transmission electron microscopy was used to verify the crystallinity and nature of the nanoparticles of commercial chitosan and prepared ChNPs (JEM-2100; JEOL Ltd., Akishima, Tokyo, Japan). An aqueous dispersion of the particles was drop-casted onto a carbon-coated copper grid and air-dried at ambient temperature before the examination.

### Statistical analysis

All treated samples were collected in triplicate. Data are expressed as mean ± standard error. Data were statistically analyzed by analysis of variance using Statistical Package for the Social Sciences 23 for Windows (IBM Corp., NY, USA). Multiple comparisons of means were performed using the least significant difference test at the significance level of p < 0.05.

## Results and Discussion

Although some yeast species play an important role in cheese ripening and may be used as starter cultures [6], over 60 species of yeast have been reported as spoilage agents in dairy products such as Karish cheese [35]. *Debaryomyces hansenii*, *G. candidum*, *K. marxianus*, *K. lactis*, *R. mucilaginosa*, *Candida* spp., and *Trichosporon* spp. are the most common yeast species causing cheese spoilage [5].

The presence of yeast in soft cheese is unacceptable, even at low levels, because it results in unwanted changes that lower the quality of the final product during storage [35]. Recently, in Egypt, several studies have reported a high incidence of yeast of several species in traditionally produced Karish cheese, with subsequent decreases in cheese quality, safety, and shelf-life [4, 35–41].

Yeast and mold are effectively inhibited by sorbate preservatives (E200-203), which do not alter the taste, color, or flavor of cheese, but concerns remain over their safety [12, 30]. Some consumers, especially those at high risk, may develop an allergy to sorbates. In addition, sorbates have been reported to cause chromosomal aberrations in cultured human lymphocytes. They also have the potential to generate a shortage of glycine, which can, in turn, negatively influence brain neurochemistry. Therefore, caution should be taken regarding exposure to high amounts of this agent [42, 43].

Data presented in Table-3 show that the *R. mucilaginosa* count in a control sample (CR) was significantly (p < 0.05) decreased in the 1^st^ week but then significantly increased (p < 0.05) until reaching 10.71 log_10_ CFU/g on the 21^st^ day of storage, with the appearance of signs of deterioration after 12 days. This may have been attributable to the effect of the lower storage temperature at the beginning, which was then overcome by the yeast cells.

**Table-3:** Survival of *Rhodotorula mucilaginosa* in produced Karish cheese with different treatments (log_10_ Mean ± SE).

Groups	Zero day	3^rd^ day	6^th^ day	9^th^ day	12^th^ day	15^th^ day	18^th^ day	21^st^ day
CR	9.46 ± 0.12^Ba^	6.97 ± 0.16^Cabc^	8.05 ± 0.05^Ca^	7.87 ± 0.21^Ca^	9.55 ± 0.28^ABa^ S	9.99 ± 0.08^ABa^ S	10.36 ± 0.06^ABa^ S	10.71 ± 0.56^Aa^ S
PR1	9.04 ± 0.52^Aa^	6.60 ± 0.32^Bbc^	5.56 ± 0.17^Ce^	4.75 ± 0.15^Cb^	4.40 ± 0.53^Cb^	3.81 ± 0.06^CDc^	2.72 ± 0.15^Dc^	ND
PR2	8.77 ± 0.72^Aa^	5.89 ± 0.04^Bcd^	4.93 ± 0.12^Bde^	4.72 ± 0.42B^Cbc^	3.28 ± 0.24^CDbc^	2.39 ± 0.2^Dd^	ND	ND
CR1	9.13 ± 0.29^Aa^	7.77 ± 0.03^Ba^	7.67 ± 0.12^Ba^	4.59 ± 0.06^Cb^	4.03 ± 0.1^Cb^	4.63 ± 0.17^CDb^	3.67 ± 0.1^Db^	2.54 ± 0.16^Eb^
CR2	9.10 ± 0.28^Aa^	7.66 ± 0.39^Ba^	6.51 ± 0.26^Bb^	4.86 ± 0.09^Cb^	4.27 ± 0.17^CDb^	4.70 ± 0.07^Dc^	2.51 ± 0.26^Ec^	ND
NR1	9.56 ± 0.71^Aa^	7.32 ± 0.36^Bab^	5.81 ± 0.07^Cbc^	3.71 ± 0.13^Dbc^	3.33 ± 0.07^Dbc^	2.40 ± 0.2^Dd^	ND	ND
NR2	9.51 ± 0.26^Aa^	6.60 ± 0.15^Bbc^	5.51 ± 0.26^Ccd^	3.78 ± 0.06^Dc^	2.75 ± 0.14^Ec^	ND	ND	ND
MR	8.92 ± 0.55^Aa^	4.82 ± 0.06^Bd^	2.65 ± 0.18^Cf^	ND	ND	ND	ND	ND

CR=Control Karish cheese with Rhodotorula, PR1=Karish cheese with 0.1% potassium sorbate, PR2=Karish cheese with 0.2% potassium sorbate, CR1=Karish cheese with 1% chitosan, CR2=Karish cheese with 2% chitosan, NR1=Karish cheese with 0.25% nano-chitosan, NR2=Karish cheese with 0.5% nano-chitosan, MR=Karish cheese with 0.1% potassium sorbate and 2% chitosan, S=Spoilage signs appearance, ND=Not detected. Mean values with different lowercase letters superscripts within same column are significantly (p <0.05) different. Mean values with different uppercase letters superscripts within same raw are significantly (p < 0.05) different. SE=Standard error.

With regard to the effect of potassium sorbate, it was observed that, in treatments PR1 and PR2, the *Rhodotorula* count was significantly (p < 0.05) decreased to 6.60 log_10_ CFU/g and 5.89 log_10_ CFU/g on the 3^rd^ day, respectively, compared with that on CR treatment (6.97 log_10_ CFU/g). In addition, *Rhodotorula* was not detected on the 21^st^ and 18^th^ days of storage in treatments PR1 and PR2, respectively (Table-3). These results agree with those obtained in previous studies by Awaad *et al*. [12], Alrabadi *et al*. [44], Musyoka *et al*. [45], Elsharawy *et al*. [46], in which potassium sorbate was recorded as a preservative that inhibits the growth of yeast compared with the findings in the control. Sorbates most likely prevent microbial development by altering the morphology and function of cell membranes, and inhibiting transport functions and metabolic activity [30].

Meanwhile, chitosan showed an antifungal effect against *R. mucilaginosa*, but with a lower effect than potassium sorbate. Table-3 indicates that the use of chitosan at a higher concentration (CR2) had a better effect than CR1, which did not differ from the control group in the first 6 days. The count of *Rhodotorula* was significantly (p < 0.05) decreased using CR2 after 6 days of storage (6.51 log_10_ CFU/g) compared with that in the control group (8.05 log_10_ CFU/g), but still significantly lower than that using PR1 (5.56 log_10_ CFU/g). By the end of the storage period, *R. mucilaginosa* became uncountable in all treatments, except for CR1, with which it was only lowered to 2.54 log_10_ CFU/g but without the appearance of red pigment These results are incompatible with the previous reportsby El-Diasty *et al*. [14], Elsharawy *et al*. [46] describing that treatment of Karish cheese by adding chitosan suppressed mold and yeast growth and prolonged the cheese shelf-life.

The antimicrobial action of chitosan against microorganisms was in the following order of intensity: yeasts > molds > Gram-positive bacteria > Gram-negative bacteria [47]. The MW of chitosan varies from 50 kDa to 1000 kDa, with a DD of 30%–95%, depending on the source and method of treatment. Both MW and DD determine the properties and mode of action of chitosan in biological systems. To improve the chitosan kinetics, bioavailability, and stability, it was recently used in the form of ChNPs. Nanosized materials frequently exhibit improved properties compared to the base materials they derive [48].

Table-3 indicates that ChNPs with low concentrations showed a significantly greater antifungal effect than chitosan material with its regular MW. The addition of ChNPs at either 0.25% (NR1) or 0.5% (NR2) during Karish cheese manufacturing significantly decreased the *Rhodotorula* count. This decline was significantly greater than for chitosan treatments (CR1 and CR2), but smaller than for potassium sorbate until day 6. After 9 days of storage, NR2 treatment showed a more significant decrease in the count (3.78 log_10_ CFU/g) than even PR2 (4.72 log_10_ CFU/g), indicating the better stability of ChNPs. The count decreased to zero after 15 days of storage using NR2 and 18 days of storage using NR1.

The supplementation and coating of cheese with nano-chitosan have been reported to prevent the contamination and growth of yeast for a longer period than in control groups not coated with ChNPs [49].

Table-4 presents the antifungal effects of the same agents against *C. albicans*. The results clearly support the results in Table-3 regarding the antifungal effects of potassium sorbate, chitosan, and ChNPs. There was a significant (p < 0.05) reduction in *C. albicans* count starting from the 6^th^ day with PC1 (8.15 log_10_ CFU/g) and PC2 treatments (5.79 log_10_ CFU/g), compared with that with the CC treatment (9.89 log_10_ CFU/g) on the same day. *Candida* *albicans* count was significantly (p < 0.05) decreased in PC1 and PC2 treatments throughout the storage period, until it decreased to zero on the 15^th^ and 12^th^ days, respectively. Meanwhile, in CC treatment, the count was significantly (p < 0.05) increased throughout the storage period until it reached 12.08 log_10_ CFU/g on the 21^st^ day with the appearance of signs of deterioration after 15 days of storage. These results are in line with a previous study by Stanojevic *et al*. [50 ] showing that potassium sorbate is effective against *C. albicans*.

**Table-4:** Survival of *Candida albicans* in produced Karish cheese with different treatments (log_10_ Mean ± SE).

Groups	Zero day	3^rd^ day	6^th^ day	9^th^ day	12^th^ day	15^th^ day	18^th^ day	21^st^day
CC	10.54 ± 0.62^ABa^	9.93 ± 0.45^ABa^	9.89 ± 0.58^Ba^	10.35 ± 0.27^ABa^	12.04 ± 0.26^Aa^	11.96 ± 0.04^Aa^ S	12.18 ± 0.27^Aa^ S	12.08 ± 0.43^Aa^ S
PC1	9.98 ± 0.40^Aa^	9.24 ± 0.31^ABa^	8.15 ± 0.29^Bab^	4.26 ± 0.63^Cbc^	3.49 ± 0.24^Cc^	ND	ND	ND
PC2	9.63 ± 0.64^Aa^	7.79 ± 0.61^ABab^	5.79 ± 0.65^Bbc^	3.16 ± 0.58^Cc^	ND	ND	ND	ND
CC1	10.37 ± 0.40^Aa^	8.66 ± 0.35^ABa^	7.20 ± 0.25^Bb^	5.63 ± 0.57^Cb^	4.79 ± 0.44^Cbc^	4.66 ± 0.37^Cb^	2.66 ± 0.37^Db^	ND
CC2	10.20 ± 0.25^Aa^	8.61 ± 0.63^ABa^	7.31 ± 0.81^BCab^	4.82 ± 0.24^Dbc^	5.43 ± 0.50^CDb^	3.98 ± 0.17^DEb^	ND	ND
NC1	10.37 ± 0.40^Aa^	8.70 ± 0.65^ABa^	7.41 ± 0.53^Bab^	5.10 ± 0.61^Cbc^	4.12 ± 0.11^CDbc^	2.73 ± 0.29^Dc^	ND	ND
NC2	10.42 ± 0.39^Aa^	8.63 ± 0.57^Ba^	7.05 ± 0.21^Bb^	4.93 ± 0.37^Cbc^	3.66 ± 0.37^CDc^	2.82 ± 0.34^Dc^	ND	ND
MC	9.57 ± 0.31^Aa^	5.77 ± 0.55^Bb^	4.28 ± 0.17^Cc^	ND	ND	ND	ND	ND

CC=Control Karish cheese with *Candida albicans*; PC1=Karish cheese with 0.1% potassium sorbate; PC2=Karish cheese with 0.2% potassium sorbate; CC1=Karish cheese with 1% chitosan; CC2=Karish cheese with 2% chitosan; NC1=Karish cheese with 0.25% nano-chitosan; NC2=Karish cheese with 0.5% nano-chitosan; MC=Karish cheese with 0.1% potassium sorbate and 2% chitosan. S=spoilage signs appearance. ND=Not detected. Mean values with different lowercase letters superscripts within same column are significantly (p <0.05) different. Mean values with different uppercase letters superscripts within same raw are significantly (p < 0.05) different. SE=Standard error

The results clearly showed that chitosan has a good antifungal effect against *C. albicans*, however, its effects are still weaker than that of potassium sorbate even at a high concentration (2%). There were significant reductions in the count of *C. albicans* starting from the 6^th^ day of storage to 7.20 log_10_ CFU/g and 7.31 log_10_ CFU/g for CC1 and CC2, respectively, compared with the findings for CC (9.89 log_10_ CFU/g) and PC1 (8.15 log_10_ CFU/g). *Candida albicans* was completely eliminated after 21 days and 18 days of storage at refrigerator temperature in Karish cheese samples containing CC1 and CC2, respectively, which are longer periods than for PC1 and PC2 (15 and 12 days, respectively) (Table-4).

In a previous study by Peña *et al*. [51], chitosan was recommended to be used at concentrations higher than 1.0 mg/mL to ensure a fungicidal, not only a fungistatic, effect on *C. albicans*. In addition, the minimum inhibitory concentrations for chitosan against *C. albicans* previously reported by Tsai *et al*. [52], Balicka-Ramisz *et al*. [53], Hongpattarakere and Riyaphan [54] to be 500, 600, and >1250 ppm.

The usage of nanoparticles is widespread in different industries, including the food industry, as nanosized particles have a higher potential [55]. The addition of ChNPs to Karish cheese significantly reduced the count of *C. albicans* from the 6^th^ day of storage compared with that in the control group. In addition, the effect of ChNPs was significantly greater than that of chitosan at a high concentration, as the *C. albicans* counts were significantly lowered at day 12 of storage after using NC2 (3.66 log_10_ CFU/g) and NC1 (4.12 log_10_ CFU/g), compared with that for CC2 (5.43 log_10_ CFU/g). The count was zero after 18 days of storage in both treatments (Table-4). The findings on antifungal activity in the current study agree with those reported previously by Ing *et al*. [56], indicating that the ChNPs are natural antifungal agents against *C. albicans*.

Potassium sorbates are safe for humans at the permitted dosage, but they are widely used in the food industry, and consuming large amounts may result in certain health issues [5, 57]. Therefore, to improve the antifungal effect of chitosan and reduce the amount of potassium sorbate used, a mixture of 0.1% potassium sorbate and 2% chitosan was applied (MR and MC). This mixture showed better antifungal effects against both *R. mucilaginosa* and *C. albicans* than the other treatments, as indicated in Tables-3 and 4. This combination reduced the counts of *R. mucilaginosa* and *C. albicans* from 8.92 and 9.57 log_10_ CFU/g in MR and MC treatments, respectively, to become undetectable on the 9^th^ day of storage, which was earlier than for all the other treatments. The antifungal effect of that mixture was strongly evident, with a more significant decrease in count than for other treatments from the 3^rd^ day of storage of 4.82 and 5.77 log_10_ CFU/g for MR and MC, respectively. The value of such a combination was supported by a previous study by Fajardo *et al*. [58], which demonstrated that the combination of chitosan and natamycin exerted better inhibitory effects on mold and yeast in cheese than their use alone (Tables-3 and 4).

The pH of the product affects the preservative’s effectiveness [50]. For example, potassium sorbate’s antimicrobial action depends on the undissociated form of its molecule, which is most effective when used below pH 6.5 [59, 60]. In addition, the growth and survival of microorganisms during processing and storage are influenced by pH [61] and processors depend on the regulation of food pH as an important measure in product spoiling control [62].

Tables-5 and 6 show the difference in pH between different treatments. The results reveal that, immediately after cheese curdling and before storage, the pH values differed significantly (p < 0.05), with the highest values recorded for Karish cheese samples with 2% chitosan (CR2 and CC2: 6.2 and 6.3, respectively), which improved the antibacterial effect of chitosan as it retards the bacterial starter culture and acid production as in control samples (CR and CC: 5 for each).

**Table-5:** pH values of produced Karish cheese with different treatments inoculated with *Rhodotorula mucilaginosa* (Mean ± SE).

Groups	0 day	3^rd^ day	6^th^ day	9^th^ day	12^th^ day	15^th^ day	18^th^ day	21^st^day
CR	5 ± 0.02^Ad^	4.8 ± 0^ABf^	4.8 ± 0.03^ABf^	4.7 ± 0.04^BCc^	4.7 ± 0.04^BCc^	4.6 ± 0.05^BCc^	4.5 ± 0.08^Cbc^	4.5 ± 0.02^Cbc^
PR1	5.7 ± 0.07^Abc^	5.4 ± 0.01^Bcd^	5.3 ± 0.01^BCd^	5.2 ± 0^CDb^	5 ± 0.04^DEb^	4.9 ± 0.03^Eb^	4.7 ± 0.02^Fb^	4.6 ± 0.01^Fab^
PR2	5.9 ± 0.02^Ab^	5.8 ± 0.01^ABb^	5.7 ± 0^ABCb^	5.7 ± 0.05^ABa^	5.6 ± 0.02^BCa^	5.5 ± 0.05^Ca^	5 ± 0.03^Da^	4.8 ± 0.04^Ea^
CR1	5.7 ± 0.02^Ab^	5.5 ± 0.04^ABc^	5.5 ± 0.05^ABc^	5.2 ± 0.01^BCb^	5.1 ± 0.1^CDb^	5.3 ± 0.02^BCa^	5.1 ± 0.05^CDa^	4.7 ± 0.1^Eab^
CR2	6.2 ± 0.1^Aa^	6.1 ± 0.06^Aa^	6.2 ± 0.02^Aa^	5.8 ± 0.04^Ba^	5.6 ± 0.03^BCa^	5.3 ± 0.02^Ca^	5 ± 0.06^Da^	4.8 ± 0.03^Da^
NR1	5.1 ± 0.06^Ad^	5.1 ± 0.05^ABe^	4.9 ± 0.06^ABCef^	4.8 ± 0^BCc^	4.7 ± 0.03^CDc^	4.6 ± 0.03^DEc^	4.6 ± 0.03^DEb^	4.5 ± 0.02^Ebc^
NR2	5.2 ± 0.02^Ad^	5.1 ± 0.06^ABe^	5 ± 0.05^BCef^	4.8 ± 0.03^CDc^	4.8 ± 0.02^CDEbc^	4.6 ± 0.04^DEFc^	4.5 ± 0.05^EFbc^	4.5 ± 0.04^Fbc^
MR	5.3 ± 0.03^Acd^	5.3 ± 0.04^Ade^	5 ± 0.03^Be^	4.8 ± 0.04^Cc^	4.7 ± 0.02^Cc^	4.4 ± 0.03^Dc^	4.3 ± 0.03^Dc^	4.3 ± 0.03^Dc^

CR=Control Karish cheese with *Rhodotorula*; PR1=Karish cheese with 0.1% potassium sorbate; PR2=Karish cheese with 0.2% potassium sorbate; CR1=Karish cheese with 1% chitosan; CR2=Karish cheese with 2% chitosan; NR1=Karish cheese with 0.25% nano-chitosan; NR2=Karish cheese with 0.5% nano-chitosan; MR=Karish cheese with 0.1% potassium sorbate and 2% chitosan. Mean values with different lowercase letters superscripts within same column are significantly (p < 0.05) different. Mean values with different uppercase letters superscripts within same raw are significantly (p < 0.05) different. SE=Standard error.

**Table-6:** pH values of produced Karish cheese with different treatments inoculated with *Candida albicans* (Mean ± SE).

Groups	0 day	3^rd^ day	6^th^ day	9^th^ day	12^th^ day	15^th^ day	18^th^ day	21^st^day
CC	5 ± 0^Ad^	4.9 ± 0.01^Af^	4.9 ± 0.01^Ac^	4.8 ± 0.01^Bbc^	4.8 ± 0^Bb^	4.8 ± 0.01^Bde^	4.6 ± 0.03^Cd^	4.5 ± 0.01^Cb^
PC1	5.5 ± 0.1^Bc^	5.6 ± 0.03^ABbc^	5.8 ± 0.03^Aa^	5.7 ± 0.01^ABa^	5.5 ± 0.01^Ba^	5.1 ± 0^Cb^	4.7 ± 0.01^Dbc^	4.5 ± 0.02^Db^
PC2	5.8 ± 0^Ab^	5.8 ± 0.05^ABb^	5.9 ± 0.05^Aa^	5.7 ± 0.02^ABa^	5.6 ± 0.01^Ba^	5.4 ± 0.01^Ca^	5.3 ± 0.01^Ca^	5 ± 0.04^Da^
CC1	5.6 ± 0^Abc^	5.4 ± 0.01^ABcd^	5.3 ± 0.07^Bb^	4.9 ± 0.04^Cb^	5 ± 0.01^Cb^	4.9 ± 0.01^CDc^	4.8 ± 0.01^CDb^	4.7 ± 0^Db^
CC2	6.3 ± 0.1^Aa^	6.2 ± 0.08^Aa^	5.9 ± 0^ABa^	5.6 ± 0.1^Ba^	5.5 ± 0.1^BCa^	5.3 ± 0.03^Ca^	5.2 ± 0.04^CDa^	5.1 ± 0.1^Da^
NC1	5.3 ± 0.04^Ac^	5.2 ± 0.02^ABde^	5.1 ± 0.06^Bbc^	4.9 ± 0.03^Cb^	4.9 ± 0.03^Cb^	4.8 ± 0^CDcd^	4.7 ± 0.01^CDbc^	4.7 ± 0^Db^
NC2	5.3 ± 0.02^Ac^	5.2 ± 0.05^ABdef^	5 ± 0.1^BCc^	4.9 ± 0.05^CDbc^	4.8 ± 0^CDb^	4.8 ± 0.02^CDde^	4.7 ± 0.01^CDbc^	4.7 ± 0.01^Db^
MC	5.2 ± 0.01^Acd^	5 ± 0.1^ABef^	4.9 ± 0.01^BCc^	4.9 ± 0.01^BCb^	4.9 ± 0.01^BCDb^	4.7 ± 0^CDEe^	4.6 ± 0.04^DEcd^	4.5 ± 0.01^Eb^

CC=Control Karish cheese with *Candida albicans*, PC1=Karish cheese with 0.1% potassium sorbate, PC2=Karish cheese with 0.2% potassium sorbate, CC1=Karish cheese with 1% chitosan, CC2=Karish cheese with 2% chitosan, NC1=Karish cheese with 0.25% nano-chitosan, NC2=Karish cheese with 0.5% nano-chitosan, MC=Karish cheese with 0.1% potassium sorbate and 2% chitosan. Mean values with different lowercase letters superscripts within same column are significantly (p < 0.05) different. Mean values with different uppercase letters superscripts within same raw are significantly (p < 0.05) different. SE=Standard error.

It was also clear that the use of potassium sorbate at different concentrations (0.1% and 0.2%) greatly affected pH values to produce significantly higher levels than in the control groups (5 for both CR and CC). The values were 5.7, 5.9, 5.5, and 5.8 for PR1, PR2, PC1, and PC2, respectively, which were still significantly lower than those of CR2 (6.2) and CC2 (6.3) (Tables-5 and 6). In addition, 0.2% potassium sorbate retarded acid production by more than 0.1% throughout the storage period. Specifically, pH on the 21^st^ day of storage was significantly (p < 0.05) higher in PR2 (4.8) than in PR1 (4.6) (Table-5). Hence, the significant reductions in *R. mucilaginosa* and *C. albicans* counts could have been related directly to the effect of potassium sorbate not to the effect of pH.

A significant decline in pH was identified throughout the storage period with significant (p < 0.05) differences in all groups. By the end of the storage period (21 days), the pH differed significantly between groups, but the mixture groups (MR and MC) and nano-chitosan groups with both concentrations (NR1, NR2, NC1, and NC2) showed the highest stability, with non-significant differences from the control groups (CR and CC). Meanwhile, at the end of storage, PR2, PC2, CR2, and CC2 showed significantly high pH values among the groups (4.8, 5.0, 4.8, and 5.1, respectively) (Tables-5 and 6). The results on pH remained in agreement with those reported previously by Saleh [1].

**Figure-2:**
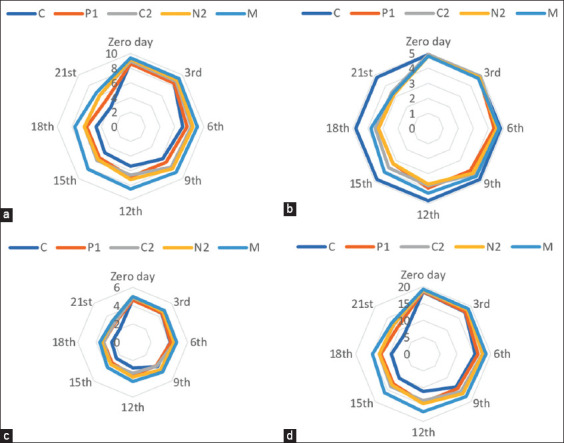
Sensory evaluation of produced Karish cheese with different treatments during storage period (21 days). (a) Flavor (10), (b) body and texture, (c) color and appearance, (d) overall (20). C=Control Karish cheese, P1=Karish cheese with 0.1% potassium sorbate, C2=Karish cheese with 2% chitosan, N2=Karish cheese with 0.5% Nano-chitosan, M=Karish cheese with 0.1% potassium sorbate and 2% chitosan.

Data presented in Table-7 and Figures-2a–d revealed no significant differences in the mean flavor, body and texture, color, and appearance in all treatments until the 6^th^ day of storage. On the 6^th^ day of storage, significantly (p < 0.05) lower values for flavor (7.1), body, and texture (4.2)were recorded for the control group.

**Table-7:** Sensory evaluation of produced Karish cheese with different treatments during storage period (21 days) (Mean ± SE).

Sensory parameters	0 day	3^rd^ day	6^th^ day	9^th^ day	12^th^ day	15^th^ day	18^th^ day	21^st^ day
Flavor (1–10)								
C	8.8 ± 0.2^Aa^	8.4 ± 0.3^Aa^	7.1 ± 0.1^Bc^	6.2 ± 0.2^BCc^	5.4 ± 0.2^CDc^	5 ± 0.1^Dc^	4.7 ± 0.1^DEc^	3.8 ± 0.1^Ec^
P1	8.6 ± 0.3^Aa^	8.4 ± 0.3^Aa^	7.7 ± 0.2^ABbc^	6.8 ± 0.3^BCbc^	6.8 ± 0.3^BCb^	6 ± 0.1^CDb^	6 ± 0.1^CDb^	4.9 ± 0.2^Db^
C2	8.9 ± 0.3^Aa^	8.8 ± 0.^3Aa^	8.3 ± 0.3^Aab^	7.8 ± 0.3^ABab^	6.6 ± 0.2^BCb^	6.5 ± 0.1^Cb^	6.3 ± 0.1^Cb^	6 ± 0.1^Ca^
N2	9.2 ± 0.2^Aa^	9 ± 0.2^Aa^	8.5 ± 0.2^ABab^	8.1 ± 0.2^Ba^	7.2 ± 0.1^Cb^	6.3 ± 0.1^Db^	6.3 ± 0.1^Db^	6 ± 0.1^Da^
M	9.4 ± 0.2^Aa^	9.3 ± 0.2^Aa^	9.1 ± 0.1^ABa^	8.8 ± 0.1^ABCa^	8.5 ± 0.1^BCa^	8.2 ± 0.1^CDa^	7.6 ± 0.2^Da^	6.6 ± 0.2^Ea^
Body and texture (1–5)								
C	4.9 ± 0.08^Aa^	4.6 ± 0.08^ABa^	4.2 ± 0.1B^Cb^	3.8 ± 0.1^Cb^	3 ± 0.2^Cc^	2.7 ± 0.2^Dc^	2.5 ± 0.2^Db^	2.3 ± 0.2^Db^
P1	4.9 ± 0.1^Aa^	4.7 ± 0.06^Aa^	4.3 ± 0.05^ABab^	3.9 ± 0.05^BCb^	4 ± 0.05^CDab^	3.3 ± 0.1^Db^	3.3 ± 0.1^Da^	3.2 ± 0.1^Da^
C2	4.9 ± 0.06^Aa^	4.9 ± 0.06^Aa^	4.6 ± 0.08^Aa^	4.3 ± 0.1^ABab^	3.8 ± 0.1^BCb^	3.7 ± 0.1^Cab^	3.5 ± 0.1^Ca^	3.4 ± 0.1^Ca^
N2	4.8 ± 0.1^Aa^	4.8 ± 0.1^Aa^	4.5 ± 0.04^Aab^	4.2 ± 0.1^ABab^	3.7 ± 0.1^BCb^	3.3 ± 0.2C^b^	3.3 ± 0.2^Da^	3.2 ± 0.1^Da^
M	4.8 ± 0.1^Aa^	4.7 ± 0.1^Aa^	4.7 ± 0.1^Aa^	4.5 ± 0.2^ABa^	4.3 ± 0.1^ABCa^	4.1 ± 0.1^BCa^	3.8 ± 0.1^CDa^	3.3 ± 0.1^Da^
Color and appearance (1–5)								
C	4.7 ± 0.1^Aa^	4.5 ± 0.2^ABa^	4.1 ± 0.2^ABb^	3.8 ± 0.1^Ba^	2.8 ± 0.2^Cc^	2.5 ± 0.2^CDb^	2.3 ± 0.2^CDb^	2 ± 0^Db^
P1	4.6 ± 0.2^Aa^	4.5 ± 0.2^Aa^	4.1 ± 0.09^ABb^	3.6 ± 0.2^BCa^	3.6 ± 0.2^BCabc^	3.2 ± 0.1^CDab^	3.2 ± 0.1^CDa^	2.8 ± 0.09^Da^
C2	4.8 ± 0.05^Aa^	4.8 ± 0.05^Aa^	4.6 ± 0.1^ABab^	3.7 ± 0.4^ABCa^	3.4 ± 0.3^BCbc^	3.4 ± 0.3^BCab^	3.2 ± 0.3^Ca^	2.9 ± 0.2^Ca^
N2	5 ± 0^Aa^	4.7 ± 0.1^ABa^	4.6 ± 0.1^ABab^	4.3 ± 0.1^BCa^	3.8 ± 0.1^CDab^	3.4 ± 0.2^Dab^	3.4 ± 0.2^Da^	3.2 ± 0.1^Da^
M	5 ± 0^Aa^	4.9 ± 0.1^Aa^	4.8 ± 0.1^ABa^	4.6 ± 0.2^ABa^	4.3 ± 0.1^BCa^	3.9 ± 0.1^CDa^	3.6 ± 0.1^DEa^	3.2 ± 0.1^Ea^
Overall (100)								
C	92 ± 1.4^Aab^	87 ± 1.8^Ab^	77 ± 0.9^Bc^	67 ± 1.4^Cd^	55 ± 0.9^Dc^	50 ± 0.9^DEc^	47 ± 1.2^Ec^	40 ± 0^Fc^
P1	91 ± 1.6^Ab^	88 ± 1.8^ABb^	82 ± 1.2^Bc^	72 ± 2^Ccd^	72 ± 2^Cb^	62 ± 1.1^Db^	62 ± 1.1^Db^	54 ± 1.5^Eb^
C2	94 ± 1.3^Aab^	93 ± 1.5^Aab^	87 ± 1.7^Ab^	79 ± 2.2^Bbc^	69 ± 1.7^Cb^	68 ± 1.7^Cb^	65 ± 2^Cb^	61 ± 1.2^Ca^
N2	95 ± 0.8^Aab^	93 ± 1.1^ABab^	88 ± 1.2^BCab^	83 ± 1.2^Cab^	74 ± 1.3^Db^	65 ± 2^Eb^	65 ± 2^Eb^	62 ± 1.2^Ea^
M	96 ± 0.6^Aa^	95 ± 0.9^Aa^	93 ± 0.9^ABa^	89 ± 1.5^BCa^	85 ± 0.9^CDa^	81 ± 1^Da^	75 ± 1.1^Ea^	66 ± 1.2^Fa^

C=Control Karish cheese, P1=Karish cheese with 0.1% potassium sorbate, C2=Karish cheese with 2% chitosan, N2=Karish cheese with 0.5% nano-chitosan, M=Karish cheese with 0.1% potassium sorbate and 2% chitosan. Mean values with different lowercase letters superscripts within same column are significantly (p < 0.05) different. Mean values with different uppercase letters superscripts within same raw are significantly (p < 0.05) different. SE=Standard error.

The overall acceptability was significantly lower in the P1 group from the day of production (91) than in the other treatments, with the M group showing the highest significant acceptability (96). This difference was noticeable in the overall score between treatments on the 3^rd^ day of examination, with the highest score being found in the M group (95) and the lowest one in the C and P1 groups (87 and 88, respectively) (Figure-2d). After 9 days of storage, this variation became more pronounced and it could be attributed to the significantly lower values in flavor, body and texture, color, and appearance in the C and P1 groups than in the M group (Table-7 and Figure-2a–d).

Although all sensorial attributes significantly declined throughout the storage period, the acceptability score was higher in treated Karish cheese samples than in the control group, and the sensory properties changed with a loss of acceptability among consumers. Therefore, the addition of potassium sorbate, chitosan, and nano-chitosan either alone or in combination significantly improved the taste, body, texture, color, appearance, and overall acceptability of treated cheese throughout the storage period. By the end of storage, the highest overall acceptability was found in the M, N2, and C2 groups, with no significant difference (66, 62, and 61, respectively), followed by the P1 group (54) with a significant difference from the C group (40) (Figure-2d).

The current results are in accordance with the previous studies by El-Diasty *et al*. [14], Sayed-Elahl *et al*. [49], Mehyar *et al*. [63] that reported that cheese treated with chitosan (0.5% and 1%) showed an improvement of its sensorial quality up to the 18^th^ day of storage, while in the control group, changes of taste and texture of cheese were observed on the 6^th^ day, while variations in color appeared by the 9^th^ day.

In the previous studies by Awaad *et al*. [12], Babacan and Özdemir [64], it was also concluded that there was no adverse effect of potassium sorbate on the sensorial quality of cheese, which could be attributed to its mild effect on catalase-negative bacteria (lactic acid bacteria).

## Conclusion

Karish cheese is a highly perishable food traditionally produced from raw milk, which increases the incidence of yeast with subsequent product loss due to rapid spoilage, even in a refrigerator, and potential health risks. Therefore, the addition of an antifungal agent as a preservative is necessary. Our study concluded that potassium sorbate, chitosan, and ChNPs are effective antifungal preservatives against *R. mucilaginosa* and *C. albicans*, although *C. albicans* was more sensitive to the different treatments than *R*. *mucilaginosa*. These additives could prolong cheese shelf-life without affecting the sensorial attributes of Karish cheese. Meanwhile, they preserve the sensorial criteria for a longer period than in cheese without preservatives. In addition, the combination of chitosan with potassium sorbate showed synergistic antifungal activity with a smaller effect on pH and the best effect on sensorial properties.

## Authors’ Contributions

ABA, SSA, and AO: Conceptualized and designed the study and conducted the laboratory experiments. MAS and SMM: Contributed to sample and antifungal agent preparation and reviewed the manuscript. ABA, SSA, and AO: Data analysiss and drafted and revissed the manuscript. All authors have read, reviewed, and approved the final manuscript.
